# Unravelling the Molecular Identification and Antifungal Susceptibility Profiles of *Aspergillus* spp. Isolated from Chronic Pulmonary Aspergillosis Patients in Jakarta, Indonesia: The Emergence of Cryptic Species

**DOI:** 10.3390/jof8040411

**Published:** 2022-04-16

**Authors:** Anna Rozaliyani, Asriyani Abdullah, Findra Setianingrum, Wellyzar Sjamsuridzal, Retno Wahyuningsih, Anom Bowolaksono, Ayu Eka Fatril, Robiatul Adawiyah, Mulyati Tugiran, Ridhawati Syam, Heri Wibowo, Chris Kosmidis, David W. Denning

**Affiliations:** 1 Department of Parasitology, Faculty of Medicine, Universitas Indonesia, Jakarta 10430, Indonesia; findra.s88@gmail.com (F.S.); retnet2002@gmail.com (R.W.); ayufatril34@gmail.com (A.E.F.); bundaadah@gmail.com (R.A.); dramulyati@yahoo.co.id (M.T.); ridhawatia@yahoo.com (R.S.); bowoheri04@gmail.com (H.W.); 2 Indonesia Pulmonary Mycoses Centre, Jakarta 10430, Indonesia; 3 Magister Program of Biomedical Sciences, Faculty of Medicine, Universitas Indonesia, Jakarta 10430, Indonesia; asriyaniabdullah1464@gmail.com; 4 Department of Biology, Faculty of Mathematics and Natural Sciences (FMIPA), Universitas Indonesia, Depok 16424, Indonesia; sjwelly@hotmail.com (W.S.); alaksono@sci.ui.ac.id (A.B.); 5 Department of Parasitology, Faculty of Medicine, Universitas Kristen, Jakarta 13530, Indonesia; 6 Manchester Fungal Infection Group, Faculty of Biology, Medicine and Health, University of Manchester, Manchester M23 9LT, UK; chris.kosmidis@manchester.ac.uk (C.K.); ddenning@manchester.ac.uk (D.W.D.); 7 Manchester Academic Health Science Centre, Division of Infection, Immunity and Respiratory Medicine, Faculty of Biology, Medicine and Health, University of Manchester, Manchester M23 9LT, UK

**Keywords:** *Aspergillus*, cryptic, antifungal susceptibility, tuberculosis, chronic pulmonary aspergillosis

## Abstract

Cryptic species of *Aspergillus* have rapidly increased in the last few decades. Chronic pulmonary aspergillosis (CPA) is a debilitating fungal infection frequently affecting patients with previous TB. The identification and antifungal susceptibility profiles of different species of *Aspergillus* are important to support the management of CPA. The aim of this study was to describe the molecular and susceptibility profiles of *Aspergillus* isolated from CPA patients. The species identity of isolates was determined by combined DNA analyses of internal transcribed space (ITS), partial β-tubulin genes, and part of the calmodulin gene. We revealed a high (27%) prevalence of cryptic species among previous tuberculosis patients with persistent symptoms. Twenty-nine (49%) patients met the criteria for diagnosis of CPA with 24% containing *Aspergillus* cryptic species. This is the first report of five cryptic *Aspergillus* species from clinical isolates in Indonesia: *A. aculea tus*, *A. neoniger*, *A. brunneoviolacues*, *A. welwitschiae,* and *A. tubingensis*. Significantly, there was decreased sensitivity against itraconazole in the CPA group (66% susceptible to itraconazole) compared to the non-CPA group (90% susceptible to itraconazole) (*p* = 0.003). The species-level characterisation of *Aspergillus* and its antifungal susceptibility tests demands greater attention to better the management of CPA patients.

## 1. Introduction

Chronic pulmonary aspergillosis (CPA) has been one of the most common causes of persistent pulmonary symptoms found in post-tuberculosis infection patients. About three million CPA cases occur worldwide [1]. Globally, it was estimated that 1.2 million pulmonary tuberculosis cases developed into CPA [2]. In Indonesia, the prevalence of CPA is estimated at 378,700 cases [3]. Previous studies revealed around 8–56.7% patients with a history of pulmonary tuberculosis (TB) developed CPA [4,5,6]. 

*Aspergillus fumigatus* is the cause in the majority of CPA cases. However, other species have also been implicated, such as *A. flavus*, *A. niger*, *A. terreus* or *A. nidulans* [7]. The conventional methods to identify *Aspergillus* species rely on direct microscopic examination and culture to support the diagnosis of CPA [8]. However, some *Aspergillus* species are morphologically indistinguishable and molecular identification is required to identify these cryptic species [9,10,11,12]. Several genes have been used to facilitate the identification of *Aspergillus* at the species level, including internal transcribed spacer (ITS), calmodulin (CaM), and β-tubulin (benA) [13]. The increasing number of cryptic species reported worldwide indicates that these species are of concern due to the variable susceptibility profile [14,15,16]. A recent report revealed that cryptic species comprised 37% of *Aspergillus* clinical isolates [17], but whether these species play a role in the aetiology of CPA is not known. Molecular profiling of *Aspergillus* isolates in CPA has shown the presence of cryptic species in the UK [18]. Therefore, this study aims to identify the genetic profile of *Aspergillus* spp. isolated from clinical specimens of previous TB patients with suspected CPA. 

## 2. Materials and Methods 

### 2.1. Aspergillus spp. Isolates

Fifty-nine clinical isolates of *Aspergillus* were included in this study. The clinical isolates were recovered from the culture collection of the Mycology Laboratory, Department of Parasitology, Faculty of Medicine Universitas Indonesia. The sources of culture collection were the sputum of post-tuberculosis patients with suspected CPA between 2019 and 2020 obtained during routine clinical care. The diagnostic criteria of CPA are: (1) at least one of these chronic (>3 months) symptoms including haemoptysis, cough, dyspnea, chest pain and/or fatigue, and (2) positive *Aspergillus* spp. culture from sputum or positive *Aspergillus* antibodies, and (3) radiological appearances suggestive of CPA (at least fungal balls and/or cavitation confirmed by a CT scan). The study was approved by the Ethics Committee of the Faculty of Medicine, Universitas Indonesia (95/UN2.F1/ETIK/2019).

### 2.2. Molecular Identification

DNA extraction was prepared using the two-step extraction method with the precipitation reagent phenol-chloroform-isoamylalcohol as previously described with modifications [19]. The species-specific identification of all isolates was examined by amplification of the ITS rDNA gene using ITS1 (5′-TCCGTAGGTGAACCTGCGG-3′) and ITS4 (5′-TCCTCCGCTTATTGATATGC-3′) primers [20], part of the benA gene using Bt2a (5′-GGTAACCAAATCGGTGCTGCTTCT-3′) and Bt2b (5′-ACCCTCAGTGTAGTGACCCTTGGC-3′) primers [21], partial CaM gene using cmd5 (5′-CCGAGTACAAGGAGGCCTTC-3′) and cmd6 (5′-CCGATAGAGGTCATAACGTGG-3′) primers [22]. The PCR amplifications were conducted as described in detail previously with some modifications [22,23]. The results of sequencing were aligned using Mega 6.06TM followed by the basic local alignment search tool (BLAST) at The National Center for Biotechnology Information (NCBI) and the International Society for Human and Animal Mycology (ISHAM) databases. Calmodulin was used as the reference gene isolate in the *Flavi* and *Nigri* sections since beta tubulin and ITS may produce PCR biases [24,25,26,27]. 

### 2.3. Antifungal Susceptibility Tests

Antifungal susceptibility tests were performed using the disk diffusion method. Suspension of fungal colonies using a 0.9% NaCl solution was prepared with a turbidity equivalent to 0.5 of the McFarland standard. By using a sterile swab, the suspension was applied to the surface of the Muller Hinton Agar (MHA). Disk diffusion for amphotericin B (10 µg), voriconazole (1 µg) and itraconazole (8 µg disks) were obtained commercially (Liofilchem, Roseto degli Abruzzi, Italy). The plates were incubated at 35 °C for 48 h after applying the disks. The measurement of the zone of inhibition relied on a marked reduction (80%) of microcolonies after 48 h [28,29]. *Candida krusei* ATCC 6258 was used as a control strain [28,30]. The interpretations of the zone of inhibition were referenced to Espinel-Ingroff et al. [28]; the zones of inhibition of the *Candida krusei* ATCC 6258 in this study were within the reference range.

### 2.4. Statistical Analysis

Values were presented using frequencies (%) for categorical variables and means ± standard deviations and ranges for normally distributed continuous variables. The different continuous variables were analysed using an independent *t*-test for CPA and non-CPA groups or cryptic and sensu stricto groups. Fisher’s exact tests or Χ2 tests were used for categorical variables for CPA and non- CPA groups or cryptic and sensu stricto groups. Data analysis was performed with the use of IBM SPSS V.25 (IBM Corp., Armonk, NY, USA) statistic software. The significance level was set to *p* < 0.05.

## 3. Results

### 3.1. Patient Characteristics

Amongst the 59 clinical *Aspergillus* isolates from 46 patients with suspected CPA, DNA sequencing showed that 16 (27%) isolates were cryptic/rare species and 43 (73%) isolates were non-cryptic (sensu stricto) species (Table 1). Twenty-nine (49%) of the patients met the criteria for CPA, while thirty (51%) patients were diagnosed with other conditions. Seven (24%) of 29 CPA patients had cryptic *Aspergillus* isolates from their cultures. Amongst the *A. Fumigati* section, all were *A. fumigatus* sensu stricto. Likewise, most of the *Flavi* were *A. flavus* sensu stricto (88%). In contrast, most (68%) of the *A. Nigri* section was identified as cryptic species. The *Clavati* section consisted of one *A. clavatus* sensu stricto. The CPA patients had a higher rate of haemoptysis (79% vs. 43%, *p* = 0.005) and chronic haemoptysis (38% vs. 13%, *p* = 0.039) compared to the non-CPA group, which was unrelated to whether strains were or were not cryptic. 

### 3.2. Isolate Identification

The 59 isolates were morphologically classified as the *A. Fumigati* section (47%, *n* = 28), *A. Clavati* section (2%, *n* = 1), *A. Flavi* section (14%, *n* = 8), and *A. Nigri* section (37%, *n* = 22). The combination of ITS, beta tubulin, and calmodulin sequences generated in this study identified ten (four non-cryptic and six cryptic species) different species across these 59 isolates. In order of decreasing prevalence, *A fumigatus* (47%, *n* = 28), *A. flavus* (12%, *n* = 7), *A. niger* (12%, *n* = 7), *A. brunneoviolaceus* (12%, *n* = 7), *A. tubingensis* (5%, *n* = 3)*, A. aculeatus* (3%, *n* = 2)*, A. neoniger* (3%, *n* = 2), *A. clavatus* (2%, *n* = 1), *A. welwitschiae* (2%, *n* = 1) and *A. tamarii* (2%, *n* = 1) accounted for the identified isolates (Table 2). We repeated the DNA extraction and sequencing steps for the nine selected available isolates with discrepancies resulting between three primers.

### 3.3. Antifungal Susceptibility Profiles

Of the 59 isolates tested, 19% (*n* = 11) were susceptible to amphotericin B, 53% (*n* = 31) were susceptible to voriconazole, and 78% (*n* = 46) were susceptible to itraconazole based on disk diffusion tests (Table 3). Cryptic species had higher mean values of zones of inhibition to all three antifungals used in this study compared to the non-cryptic species.

Using amphotericin B, the mean values for the zone of ≥inhibition for non-cryptic and cryptic isolates were 9.7 ± 4.5 mm (range 0–22 mm) and 13.9 ± 4.6 mm (range 2–21 mm) (*p* = 0.002), respectively, indicative of cryptic species being more susceptible. In line with this, the proportion of resistant isolates in the non-cryptic group (79%) is higher (*p* = 0.002) than in the cryptic group (34%) for amphotericin B. However, the non-cryptic group (7%) showed a lower (*p* = 0.028) number of intermediate isolates against amphotericin B compared to the cryptic group (31%).

**Figure 1:**
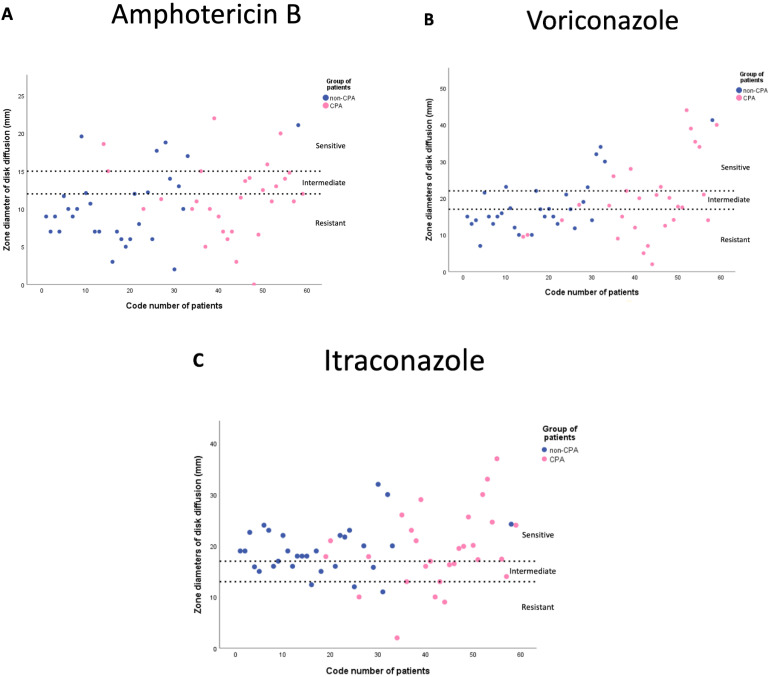
Scatter plot diagrams of zone inhibition diameters of disk diffusion against amphotericin B (**A**), voriconazole (**B**) and itraconazole (**C**) in the CPA and non-CPA groups. Zone diameter categories (dash lines): amphotericin B (susceptible ≥ 15 mm; intermediate 13 to 14 mm; resistant ≤ 12 mm), itraconazole and voriconazole (susceptible ≥ 17 mm; intermediate 14 to 16 mm; resistant ≤ 13 mm) (15).

Voriconazole revealed higher (*p* < 0.005) mean values for the zone of inhibition in the cryptic group (26.4 ± 10.4) compared to the non-cryptic group (16.1 ± 7.1). Itraconazole showed higher (*p* = 0.009) mean values for the zone of inhibition in the cryptic group (22.7 ± 7.3) compared to the non-cryptic group (18.9 ± 5.3). There are no differences in antifungal susceptibility profiles based on disease classification (CPA and non-CPA), except there were a significantly lower number (*p* = 0.003) of susceptible isolates in the CPA group (66%) compared to the non-CPA group (90%) against itraconazole. The scatter plots are shown in Figure 1A–C. The zone of inhibitions of the quality control strain was within the diameter ranges of the reference.

**Figure 2:**
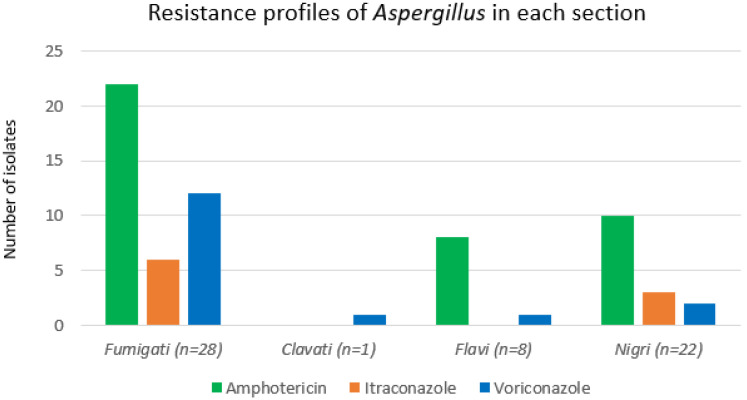
Resistance profiles of *Aspergillus* in each section. *Fumigati* and *Nigri* sections showed resistance in all three classes of antifungals (amphotericin B, voriconazole and itraconazole). There is no itraconazole resistance detected from the *Flavi* section.

**Figure 3:**
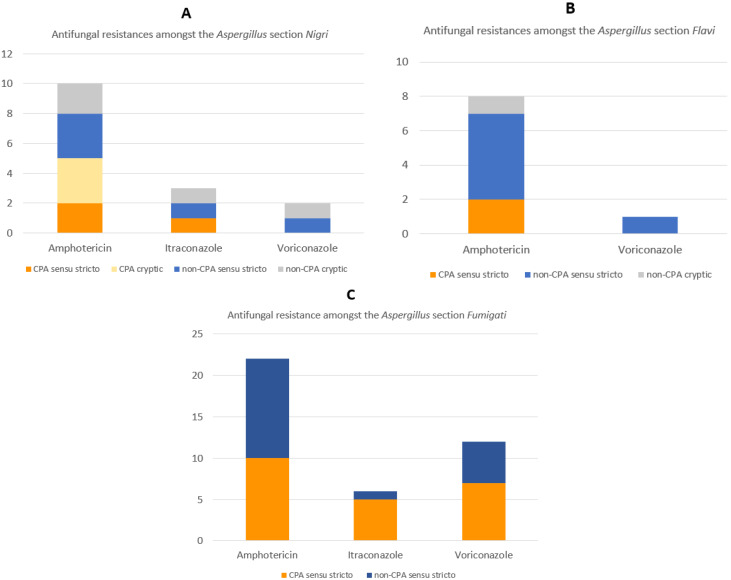
Antifungal resistances of *Aspergillus* in each section (**A**) *Flavi* section, (**B**) *Nigri* section, (**C**) *Fumigati* section and its correlation with CPA diagnosis.

Amongst the four sections of *Aspergillus* (*Fumigati*, *Clavati*, *Flavi*, and *Nigri*), the highest rate of resistance against amphotericin B was observed in the *Flavi* section (100%, *n* = 8). Meanwhile, the highest rate of resistance against itraconazole and voriconazole was seen in the *Fumigati* section (itraconazole: 21%, *n* = 6; voriconazole: 43%, *n* = 12). We excluded the *Clavati* section from these comparisons because this section only had one isolate. *A. clavatus* sensu stricto was susceptible against amphotericin and itraconazole but resistant to voriconazole (Figure 2). The *Fumigati* section showed the highest rates of resistance for azoles with 6 and 12 isolates showing resistance to itraconazole and voriconazole, respectively (Figure 3).

**Figure 4:**
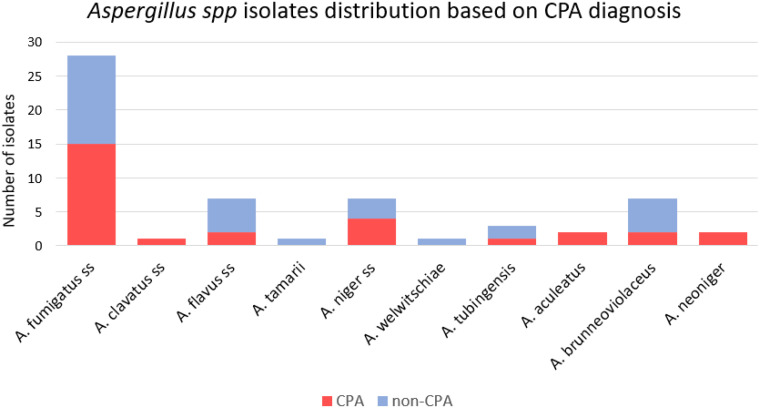
*Aspergillus* spp. isolates distribution based on chronic pulmonary aspergillosis (CPA) diagnosis.

There were 22 isolates in the *Nigri* section, consisting of 7 (32%) isolates of *A. niger* sensu stricto and 15 (68%) isolates belonging to cryptic species (Supplementary Tables S1–S4). The proportion of CPA and the non-CPA group from cryptic isolates is nearly the same. Of these 15 cryptic species isolates, there were 7 (47%) isolates from CPA patients. Meanwhile, *A. niger* sensu stricto classified as CPA was 57% (4/7) (Figure 4). Seven isolates of cryptic *Aspergillus* from the *Nigri* section classified as CPA were from *A. aculeatus* (*n* = 2), *A. neoniger* (*n* = 2), *A. tubingensis* (*n* = 1), and *A. brunneoviolaceus* (*n* = 2). Meanwhile, eight isolates were classified as the non-CPA consisting of *A. welwitschiae* (*n* = 1), A. tubi (*n* = 2) and *A. brunneoviolaceus* (*n* = 5).

There was no azole resistance detected from CPA from cryptic isolates compared with two isolates (*A. tubingensis* and *A. welwitschiae*) detected as resistance from the non-CPA cryptic group. Amphotericin B resistance was observed in three isolates (*A. tubingensis*, *A. aculeatus*, *A. neoniger*) from CPA cryptic isolates compared to two isolates from the non-CPA cryptic group (*A. brunneoviolaceus* and *A. tubingensis*).

## 4. Discussion

This is the first report of the clinical isolation of several cryptic species including *A. aculeatus*, *A. neoniger*, *A. brunneoviolaceus*, *A. welwitschiae*, *A. tubingensis* and *A. clavatus* from Indonesia. Several papers identified some of these cryptic isolates such as *A. brunneoviolaceus* (previously *A. fijiensis*), *A. japonicus*, *A. tubingensis*, *A. carbonarius* from the environment in Indonesia [31,32]. Twenty-seven percent of *Aspergillus* isolates in this study were classified as cryptic species. This rate is nearly the same as a multicenter study from China which revealed that 21.3% of clinical isolates of *Aspergillus* belong to cryptic species [16]. At 5-years follow-up, the mortality rate was 27% with two patients dying because of CPA related to *A. tubingiensis* and *Aspergillus sydowii* [16]. There is no previous study about cryptic species from CPA patients in Indonesia. However, a recent report showed that 15.6% of invasive aspergillosis patients were infected by cryptic isolates [12].

*Aspergillus aculeatus* is mostly found in plants; however, previous studies recovered *A. aculeatus* isolates from clinical specimens with many of them susceptible to antifungals [9,33,34,35]. Two patients with CPA and *A. aculeatus* in our study had amphotericin B resistant isolates. *A. brunneoviolaceus* has been previously described as an etiological cause of CPA [16] and we found one CPA isolate in our study. The occurrence of these cryptic species in our study revealed the diversity of fungal etiology of CPA in Indonesia.

This study revealed a discordance of molecular identification using three different primers (ITS, beta tubulin, calmodulin) in the *Nigri* section. There was a significant number of medically important strains from the *Nigri* section [18,36]. Additionally, the molecular analysis and genotyping of the *Nigri* section is difficult [36]. The *Nigri* section was dominated by the cryptic species in our study (88%) as previously report in recent study from Portugal (84%) [17]. Three isolates were identified as *A. aculeatus* by ITS and beta tubulin, while calmodulin grouped the isolates as *A. brunneoviolaceus*. One isolate was identified as *A. niger* by ITS, meanwhile beta tubulin and calmodulin grouped the isolate as *A. welwitschiae*. Finally, one isolate was identified as *A. flavus* by ITS and beta tubulin, while calmodulin grouped the isolate as *A. tamarii*. In this study we used calmodulin instead of beta tubulin and ITS for the reference gene in cases with different results of species identification in the *Flavi* and *Nigri* sections [24,25,37].

The discrepancies between ITS, beta tubulin, and calmodulin in some isolates might be explained by the existence of a paralogue of the beta tubulin gene named tubC [36,38,39]. The paralogue has different intron numbers in the *Nigri* section [38] and forms two different beta tubulin proteins in *A. aculeatus* and *A. japonicus* [38]. The isolates which contain two or three beta tubulin genes appeared in different branches of the parsimony tree [38]. Ben2f/Bt2b were recommended to be used as primers instead of Bt2a to prevent discordance in the molecular identification of the *Nigri* section [39]. Another explanation was the presence of the mixed colonies of the *Nigri* section since it was difficult to distinguish different species via microscopy. *A. flavus* and *A. tamarii* from the *Flavi* section are phenotypically very similar, making it possible to have two different species on one plate [11,12].

Two isolates (068-BT and 069-BT) were identified as *A. aculeatus* with ITS and beta-tubulin, while calmodulin showed the result as *A. brunneviolaceus.* We repeated the calmodulin sequencing after re-examination of the morphology of the fungi microscopically to exclude mixed culture cases in these two isolates. The second attempt of the calmodulin sequencing revealed both of the species as *A. aculeatus.* Recent evidence suggests that *A. brunneviolaceus* and *A. aculeatus* are genetically closely related [40,41]. Two strains of *A. brunneviolaceus* were previously identified as *A. aculeatus*, all of them coming from the same highly supported clade [40]. In addition, the MSP dendogram from MALDI-TOF MS clustered *A. brunneviolaceus* and *A. aculeatus* together while the phylogenetic tree based on calmodulin clearly separated these two species. Calmodulin is recommended to distinguish closely related species of *Aspergilli* [24,25]. Therefore, the final identification for 068-BT and 069-BT are *A. brunneviolaceus*.

One of the gold standards of antifungal susceptibility testing is CLSI broth micro-dilution [42]. This method is labour intensive and not routinely used in our centre. We used the disk diffusion method as this method is simple and shows excellent correlation (93.8–100%) with the CLSI broth microdilution based on previous studies [43,44,45]. However, the level of agreement between these methods was lower (66.7–87.5%) for amphotericin B [43,45], possibly because broth dilution is not generally as accurate as agar-based methods. The main limitation of the present study is that we did not perform CLSI or EUCAST methods to confirm the susceptibility profile findings due to resource constraints in Indonesia. A previous study showed a higher rate of amphotericin B resistance based on the disk diffusion test compared to CLSI broth micro-dilution [43].

Amphotericin B showed a higher rate of resistant isolates compared to azoles, and most of them were non cryptic isolates. All *A. flavus* isolates and 79% of *A. fumigatus* isolates were resistant to amphotericin B. Two out of seven patients with *A. flavus* resistant isolates met the criteria of CPA in our study. A previous study from Canada observed that 96.4% (*n* = 195) of *A. fumigatus* isolates developed resistance to amphotericin B [46]. The antifungal susceptibility profiles of *A. flavus* from our study were in line with a previous study, which showed that *A. flavus* was generally less susceptible to amphotericin B compared to *A. fumigatus* [47,48,49]. Goncalves et al. found 49.4% of *A. flavus* isolates to be amphotericin B resistant [47].

The rate of itraconazole resistance in this study is 10% (6/59), slightly higher than another study in CPA patients which showed 8% resistance after 12 months of itraconazole therapy [50]. Similarly, voriconazole resistance is higher (14%) in this study than another CPA study, which showed that 4% of patients developed resistance [50]. Most of the azole-resistant isolates were *A. fumigatus* sensu stricto isolates. A remarkably high number of resistant strains were detected from environmental isolates of *Aspergillus* in South East Asia [51,52,53].

Amongst 59 isolates, it was found four isolates (7%) showed resistance to all three antifungals included in this study. Three of them were *A. fumigatus* sensu stricto from three CPA patients and one from *A. niger* sensu stricto from a non-CPA patient. Although it was implied from our study that the cryptic species are more susceptible than the sensu stricto species to antifungals, we identified seven resistant isolates from cryptic species. Three cryptic isolates (*A. tubingensis*, *A. aculeatus*, and *A. neoniger*) from CPA patients showed amphotericin B resistance. Another four patients with resistant isolates were from the non-CPA groups: one *A. brunneoviolaceus* isolate was resistant to amphotericin B, one *A. tubingensis* isolate was resistant to itraconazole, one *A. welwitschiae* isolate was resistant to voriconazole and one *A. tamarii* isolate was resistant to amphotericin B. Cryptic species frequently showed less resistance to antifungals than the sensu stricto species [54,55].

Although the resistance rate of *Aspergillus* was lower in cryptic species, the clinical severity of the infections caused by these isolates were not known from our study. A previous study reported fatal invasive aspergillosis caused by a cryptic *Aspergillus* species [56]. A limitation of our study is the cryptic isolates belonged mostly to the *Nigri* section. The antifungal susceptibility profiles of other cryptic species from different sections of *Aspergillus* other than the *Nigri* section might indicate different results.

Data on the antifungal susceptibility of any clinical isolates of fungi in Indonesia are very scarce. This is the first study reporting the antifungal susceptibility profile from CPA patients in Indonesia. This study showed reduced susceptibility of CPA isolates against itraconazole. This finding is concerning because itraconazole is a key antifungal agent for aspergillosis, although some compounds are being investigated for the development of new antifungal drug options [57,58,59]. It is likely that patients in this study never had antifungal therapy because they were suspected to have post-tuberculosis lung disease. Azole resistance can be acquired without exposure to antifungal during azole therapy but also from the environment, for example, after exposure to triazole fungicides [60,61,62]. In a large surveillance study from the Netherlands, 64% of patients with itraconazole resistance never had prior azole treatment [63]. Further study is needed to investigate the environmental *Aspergillus* isolates in Indonesia, their susceptibility profile and the presence of resistance mutations. Studies on clinical outcomes of azole treatment in CPA in Indonesia are urgently needed in order to understand the impact of the reported higher rates of azole resistance in this population.

## Figures and Tables

**Table 1:** *Aspergillus* identification according to the section of the isolates recovered and patient’s clinical features included in this study.

	All (*n* = 59)	CPA (*n* = 29)	Non-CPA (*n* = 30)	*p*-Value	Cryptic (*n* = 16)	Sensu Stricto (*n* = 43)	*p*-Value
**Section**							
*Fumigati*	28 (49%)	15 (54%)	13 (43%)	0.519	0 (0%)	28 (65%)	<0.005
*Clavati*	1 (2%)	1 (3%)	0 (0%)	0.492	0 (0%)	1 (2%)	1
*Flavi*	8 (14%)	2 (7%)	6 (20%)	0.254	1 (6%)	7 (16%)	0.427
*Nigri*	22 (37%)	11 (38%)	11 (37%)	0.920	15 (93%)	7 (16%)	<0.005
**Symptoms**							
Haemoptysis	36 (61%)	23 (79%)	13 (43%)	0.005	12 (75%)	24 (56%)	0.236
Massive haemoptysis	19 (32%)	12 (41%)	7 (23%)	0.170	7 (44%)	12 (28%)	0.348
Recurrent haemoptysis	15 (25%)	11 (38%)	4 (13%)	0.039	3 (19%)	12 (28)	0.738

Abbreviations: CPA: chronic pulmonary aspergillosis. The grey background highlighted the cryptic and sensu stricto variables and their *p*-values.

**Table 2:** Molecular identification of all isolates.

No	Sections	Sample Code	Final ID	Genes Used for ID	Diagnosis	Amphotericin	Itraconazole	Voriconazole
1	*Fumigati*	006-BT	*A. fumigatus* sensu stricto	ITS, BenA, CaM	CPA	Resistant	Resistant	Susceptible
2		012-BT	*A. fumigatus* sensu stricto	ITS, BenA, CaM	CPA	Resistant	Susceptible	Intermediate
3		013-BT	*A. fumigatus* sensu stricto	ITS, BenA, CaM	Non-CPA	Resistant	Susceptible	Intermediate
4		014-BT	*A. fumigatus* sensu stricto	ITS, BenA, CaM	CPA	Resistant	Susceptible	Susceptible
5		015-BT	*A. fumigatus* sensu stricto	ITS, BenA, CaM	CPA	Susceptible	Susceptible	Susceptible
6		018-BT	*A. fumigatus* sensu stricto	ITS, BenA, CaM	Non-CPA	Resistant	Susceptible	Resistant
7		019-BT	*A. fumigatus* sensu stricto	ITS, BenA, CaM	Non-CPA	Resistant	Susceptible	Intermediate
8		020-BT	*A. fumigatus* sensu stricto	ITS, BenA, CaM	CPA	Resistant	Intermediate	Resistant
9		022-BT	*A. fumigatus* sensu stricto	ITS, BenA, CaM	Non-CPA	Resistant	Susceptible	Resistant
10		023-BT	*A. fumigatus* sensu stricto	ITS, BenA, CaM	CPA	Resistant	Susceptible	Susceptible
11		025-BT	*A. fumigatus* sensu stricto	ITS, BenA, CaM	Non-CPA	Resistant	Susceptible	Susceptible
12		026-BT	*A. fumigatus* sensu stricto	ITS, BenA, CaM	Non-CPA	Resistant	Susceptible	Intermediate
13		027-BT	*A. fumigatus* sensu stricto	ITS, BenA, CaM	Non-CPA	Resistant	Susceptible	Resistant
14		036-BT	*A. fumigatus* sensu stricto	ITS, BenA, CaM	Non-CPA	Resistant	Susceptible	Intermediate
15		048-BT	*A. fumigatus* sensu stricto	ITS, BenA, CaM	Non-CPA	Susceptible	Resistant	Intermediate
16		069-BT	*A. fumigatus* sensu stricto	ITS, BenA, CaM	Non-CPA	Resistant	Susceptible	Susceptible
17		080-BT	*A. fumigatus* sensu stricto	ITS, BenA, CaM	Non-CPA	Resistant	Susceptible	Resistant
18		083-BT	*A. fumigatus* sensu stricto	ITS, BenA, CaM	CPA	Resistant	Susceptible	Susceptible
19		084-BT	*A. fumigatus* sensu stricto	ITS, BenA, CaM	CPA	Susceptible	Resistant	Resistant
20		085-BT	*A. fumigatus* sensu stricto	ITS, BenA, CaM	Non-CPA	Resistant	Susceptible	Resistant
21		091-BT	*A. fumigatus* sensu stricto	ITS, BenA, CaM	CPA	Resistant	Resistant	Resistant
22		092-BT	*A. fumigatus* sensu stricto	ITS, BenA, CaM	CPA	Resistant	Resistant	Resistant
23		094-BT	*A. fumigatus* sensu stricto	ITS, BenA, CaM	CPA	Susceptible	Susceptible	Resistant
24		097-BT	*A. fumigatus* sensu stricto	ITS, BenA, CaM	CPA	Resistant	Resistant	Resistant
25		101-BT	*A. fumigatus* sensu stricto	ITS, BenA, CaM	Non-CPA	Resistant	Susceptible	Susceptible
26		103-BT	*A. fumigatus* sensu stricto	ITS, BenA, CaM	CPA	Resistant	Intermediate	Susceptible
27		109-BT	*A. fumigatus* sensu stricto	ITS, BenA, CaM	CPA	Intermediate	Intermediate	Susceptible
28		110-BT	*A. fumigatus* sensu stricto	ITS, BenA, CaM	CPA	Intermediate	Susceptible	Resistant
29	*Clavati*	064-BT	*A. clavatus* sensu stricto	ITS, BenA, CaM	CPA	Susceptible	Susceptible	Resistant
30	*Flavi*	052-BT	*A. tamarii*	CaM	Non-CPA	Resistant	Susceptible	Intermediate
31		066-BT	*A. flavus* sensu stricto	ITS, BenA, CaM	Non-CPA	Resistant	Susceptible	Susceptible
32		069-BT	*A. flavus* sensu stricto	ITS, BenA, CaM	Non-CPA	Resistant	Susceptible	Susceptible
33		071-BT	*A. flavus* sensu stricto	ITS, BenA, CaM	Non-CPA	Resistant	Susceptible	Intermediate
34		080-BT	*A. flavus* sensu stricto	ITS, BenA, CaM	Non-CPA	Resistant	Susceptible	Susceptible
35		086-BT	*A. flavus* sensu stricto	ITS, BenA, CaM	Non-CPA	Resistant	Susceptible	Resistant
36		092-BT	*A. flavus* sensu stricto	ITS, BenA, CaM	CPA	Resistant	Susceptible	Susceptible
37		103-BT	*A. flavus* sensu stricto	ITS, BenA, CaM	CPA	Resistant	Susceptible	Intermediate
38	*Nigri*	057-BT	*A. niger* sensu stricto	ITS, BenA, CaM	Non-CPA	Resistant	Resistant	Resistant
39		083-BT	*A. niger* sensu stricto	ITS, BenA, CaM	CPA	Resistant	Susceptible	Susceptible
40		064-BT	*A. niger* sensu stricto	ITS, BenA, CaM	CPA	Resistant	Resistant	Intermediate
41		074-BT	*A. niger* sensu stricto	ITS, BenA, CaM	Non-CPA	Resistant	Susceptible	Susceptible
42		079-BT	*A. niger* sensu stricto	ITS, BenA, CaM	CPA	Intermediate	Susceptible	Susceptible
43		085-BT	*A. niger* sensu stricto	ITS, BenA, CaM	Non-CPA	Resistant	Susceptible	Susceptible
44		103-BT	*A. niger* sensu stricto	ITS, BenA, CaM	CPA	Susceptible	Susceptible	Susceptible
45		076-BT	*A. welwitschiae*	BenA, CaM	Non-CPA	Susceptible	Susceptible	Resistant
46		099-BT	*A. tubingensis*	BenA, CaM	Non-CPA	Susceptible	Susceptible	Susceptible
47		101-BT	*A. tubingensis*	BenA, CaM	CPA	Resistant	Susceptible	Susceptible
48		068-BT	*A. brunneoviolaceus*	CaM	Non-CPA	Intermediate	Susceptible	Susceptible
49		073-BT	*A. aculeatus*	ITS, BenA, CaM	CPA	Intermediate	Susceptible	Susceptible
50		100-BT	*A. aculeatus*	ITS, BenA, CaM	CPA	Resistant	Susceptible	Susceptible
51		060-BT	*A. brunneoviolaceus*	CaM	CPA	Susceptible	Susceptible	Susceptible
52		006-BT	*A. brunneoviolaceus*	CaM	CPA	Intermediate	Susceptible	Susceptible
53		061-BT	*A. brunneoviolaceus*	CaM	Non-CPA	Susceptible	Susceptible	Susceptible
54		062-BT	*A. brunneoviolaceus*	CaM	Non-CPA	Intermediate	Susceptible	Susceptible
55		069-BT	*A. brunneoviolaceus*	CaM	Non-CPA	Susceptible	Susceptible	Susceptible
56		098-BT	*A. brunneoviolaceus*	CaM	Non-CPA	Resistant	Susceptible	Susceptible
57		086-BT	*A. tubingensis*	ITS, BenA, CaM	Non-CPA	Resistant	Resistant	Intermediate
58		089-BT	*A. neoniger*	CaM	CPA	Intermediate	Susceptible	Susceptible
59		097-BT	*A. neoniger*	CaM	CPA	Resistant	Intermediate	Intermediate

Abbreviations: ID: identification; CPA: chronic pulmonary aspergillosis; ITS: internal transcribed spacer; CaM: calmodulin; benA: and β-tubulin (benA).

**Table 3:** Antifungal susceptibility profiles of *Aspergillus* isolates using disk diffusion method.

	All (*n* = 59)	CPA (*n* = 29)	Non-CPA (*n* = 30)	*p*-Value	Cryptic (*n* = 16)	Sensu Stricto (*n* = 43)	*p*-Value
**Amphotericin B**							
Zone of inhibition (range)	0–22	0–22	2–21.1		2–21.1	0–22	
Mean of inhibition zone ± SD	10.8 ± 4.8	11.4 ± 4.9	10.3 ± 4.8	0.381	13.9 ± 4.6	9.7 ± 4.5	0.002
Susceptible	11 (19%)	6 (21%)	5 (17%)	0.748	5 (31%)	6 (14%)	0.149
Intermediate	8 (14%)	6 (21%)	2 (7%)	0.145	5 (31%)	3 (7%)	0.028
Resistant	40 (68%)	17 (59%)	23 (77%)	0.170	6 (38%)	34 (79%)	0.002
**Voriconazole**							
Zone of inhibition (range)	2–44	2–44	7–41.3		11.8–41.3	2–44	
Mean of inhibition zone ± SD	18.9 ± 9.2	19.6 ± 10.7	18.1 ± 7.7	0.541	26.4 ± 10.4	16.1 ± 7.1	<0.005
Susceptible	31 (53%)	17 (59%)	14 (47%)	0.358	12 (75%)	19 (44%)	0.035
Intermediate	12 (20%)	4 (14%)	8 (27%)	0.333	3 (19%)	9 (21%)	1
Resistant	16 (27%)	8 (28%)	8 (27%)	1	1 (6%)	15 (35%)	0.045
**Itraconazole**							
Zone of inhibition (range)	2–37	2–37	11–32		11–37	2–30	
Mean of inhibition zone ± SD	19.3 ± 6.2	19.3 ± 7.5	19.2 ± 4.8	0.939	22.7 ± 7.3	18 ± 5.3	0.009
Susceptible	46 (78%)	19 (66%)	27 (90%)	0.030	14 (88%)	32 (74%)	0.481
Intermediate	4 (7%)	4 (14%)	0 (0%)	0.052	1 (6%)	3 (7%)	1
Resistant	9 (15%)	6 (21%)	3 (10%)	0.299	1 (6%)	8 (19%)	0.421

Abbreviations: CPA: chronic pulmonary aspergillosis; SD: standard deviations. The grey background highlighted the cryptic and sensu stricto variables and their *p*-values.

## Data Availability

Not applicable.

## Supplementary Materials

The following supporting information can be downloaded at: https://www.mdpi.com/article/10.3390/jof8040411/s1, Table S1. The antifungal susceptibility profiles from the *Fumigati* section. Table S2. The antifungal susceptibility profiles from the *Clavati* section. Table S3. The antifungal susceptibility profiles from the *Flavi* section. Table S4. The antifungal susceptibility profiles from the *Nigri* section.
