# Supplementary material for: Unravelling the Molecular Identification and Antifungal Susceptibility Profiles of Aspergillus spp. Isolated from Chronic Pulmonary Aspergillosis Patients in Jakarta, Indonesia: The Emergence of Cryptic Species
Source: J Fungi (Basel). 2022 Apr 16;8(4):411. doi: 10.3390/jof8040411 (PMC9024953; doi:10.3390/jof8040411)
Supplement: Supplementary file 1 [file jof-08-00411-s001.zip › jof-1641933-supplementary.pdf]

Table S1. The antifungal susceptibility profiles from the *Fumigati* section.

Section *Fumigati*	Total	Amphotericin						Itraconazole						Voriconazole					
		Sensitive		Intermediate		Resistant		Sensitive		Intermediate		Resistant		Sensitive		Intermediate		Resistant	
		CPA	non-CPA	CPA	non-CPA	CPA	non-CPA	CPA	non-CPA	CPA	non-CPA	CPA	non-CPA	CPA	non-CPA	CPA	non-CPA	CPA	non-CPA
A. fumigatus sensu stricto	28	3	1	2	0	10	12	7	12	3	0	5	1	7	3	1	5	7	5

Table S2. The antifungal susceptibility profiles from the *Clavati* section.

Section *Clavati*	Total	Amphotericin						Itraconazole						Voriconazole					
		Sensitive		Intermediate		Resistant		Sensitive		Intermediate		Resistant		Sensitive		Intermediate		Resistant	
		CPA	non-CPA	CPA	non-CPA	CPA	non-CPA	CPA	non-CPA	CPA	non-CPA	CPA	non-CPA	CPA	non-CPA	CPA	non-CPA	CPA	non-CPA
A. clavatus sensu stricto	1	1	0	0	0	0	0	1	0	0	0	0	0	0	0	0	0	1	0

Table S3. The antifungal susceptibility profiles from the *Flavi* section.

Section *Flavi*	Total	Amphotericin						Itraconazole						Voriconazole					
		Sensitive		Intermediate		Resistant		Sensitive		Intermediate		Resistant		Sensitive		Intermediate		Resistant	
		CPA	non-CPA	CPA	non-CPA	CPA	non-CPA	CPA	non-CPA	CPA	non-CPA	CPA	non-CPA	CPA	non-CPA	CPA	non-CPA	CPA	non-CPA
A. flavus sensu stricto	7	0	0	0	0	2	5	2	5	0	0	0	0	1	3	1	1	0	1
A. tamarii	1	0	0	0	0	0	1	0	1	0	0	0	0	0	0	0	1	0	0

Table S4. The antifungal susceptibility profiles from the *Nigri* section.

**Section Nigri**		Total	Amphotericin						Itraconazole						Voriconazole					
			Sensitive		Intermediate		Resistant		Sensitive		Intermediate		Resistant		Sensitive		Intermediat e		Resistant	
			CPA	non-CPA	CPA	non-CPA	CPA	non-CPA	CPA	non-CPA	CPA	non-CPA	CPA	non-CPA	CPA	non-CPA	CPA	non-CPA	CPA	non-CPA
A. niger sensu stricto		7	1	0	1	0	2	3	3	2	0	0	1	1	3	2	1	0	0	1
Cryptic (n = 15)	*A. welwitschiae*	1	0	1	0	0	0	0	0	1	0	0	0	0	0	0	0	0	0	1
	A. tubingensis	3	0	1	0	0	1	1	1	1	0	0	0	1	1	1	0	1	0	0
	A. aculeatus	2	0	0	1	0	1	0	2	0	0	0	0	0	2	0	0	0	0	0
	A. brunneoviolaceus	7	1	2	1	2	0	1	2	5	0	0	0	0	2	5	0	0	0	0
	A. neoniger	2	0	0	1	0	1	0	1	0	1	0	0	0	1	0	1	0	0	0
